# New Challenges in Tribology: Wear Assessment Using 3D Optical Scanners

**DOI:** 10.3390/ma10050548

**Published:** 2017-05-18

**Authors:** Maria Cristina Valigi, Silvia Logozzo, Saverio Affatato

**Affiliations:** 1 Department of Engineering, University of Perugia, Via G. Duranti, 1, 06125 Perugia, Italy; mariacristina.valigi@unipg.it; 2 Department of Research and Development, V-GER. S.r.l., Via Mori, 6, 40054 Prunaro di Budrio (BO), Italy; 3 Medical Technology Laboratory, Rizzoli Orthopaedic Institute, Via di Barbiano, 1/10, 40136 Bologna, Italy; affatato@tecno.ior.it

**Keywords:** digital tribology, 3D optical wear measurements, 3D optical metrology and diagnostics, 3D optical scanners, wear maps

## Abstract

Wear is a significant mechanical and clinical problem. To acquire further knowledge on the tribological phenomena that involve freeform mechanical components or medical prostheses, wear tests are performed on biomedical and industrial materials in order to solve or reduce failures or malfunctions due to material loss. Scientific and technological advances in the field of optical scanning allow the application of innovative devices for wear measurements, leading to improvements that were unimaginable until a few years ago. It is therefore important to develop techniques, based on new instrumentations, for more accurate and reproducible measurements of wear. The aim of this work is to discuss the use of innovative 3D optical scanners and an experimental procedure to detect and evaluate wear, comparing this technique with other wear evaluation methods for industrial components and biomedical devices.

## 1. Introduction

Wear is a phenomenon implying a progressive loss of material of two bodies in relative motion, with consequent modification of the contact surfaces. Wear is probably the most important aspect of tribology and it remained partially unexplored until the last century. The study and monitoring of wear progression is a topic of great significance since the result of the analysis of surfaces’ modifications can lead to the definition of optimized profiles in terms of wear resistance and useful-life. However, it is difficult to assess the surface topography of an object with a complex shape—as for example teeth, knees, bones, or freeform mechanical components—due to the various irregularities and geometric configurations that are unique for each component [1].

The control and limitation of wear in industrial components is the goal in many applications to improve efficiency and reduce costs due to production, maintenance, or replacement. For example, the prediction of wear progression is useful in establishing a convenient maintenance schedule in industrial applications. In [2], the wear of the middle plate of a coal conveyor chute was evaluated to provide a reliable theoretical and technical basis for the design, use, and maintenance of the middle plate, chute, and the entire scraper conveyor; in [3], the wear resistance of a tribological contact in artificially fabricated surface textures of tribo-pairs was assessed to increase the understanding of the wear behavior of tribo-pairs between textured stainless steel and polymer materials; in [4,5], wear analyses were performed to study the alteration of the wheel-rail interface and to predict wear progression. This is a key element for reducing railroad costs, improving safety and lifespan.

Furthermore, the effort to increase the durability of medical devices has a two-fold aim: reducing the wear of bearing materials (and the formation and consequent release of small particles into the biological environment) and improving the implant design, manufacturing, and operative techniques, with the aim of minimizing the occurrence of wear. For example, in [6] another important application of wear evaluation was performed. The aim was to assess the wear behavior of dental materials and to correlate parameters with their clinical performance. 

At present, a variety of digital wear measurement methods are known and, as reported in [7], they can be classified as direct and indirect. Direct methods are referred to those applications when it is possible to directly access the worn surface optically or by contact. Usual direct measuring methods are [8]:

- Weighing [9]: according to this method, the components under study are weighed before and after a certain period of use to evaluate the mass wear rate. The difference of weight is useful also to assess the amount of the worn volume, when the exact density of the material is known and when the material is supposed to be homogenous. In this case, accurate mass balances should be used, as loss of material in wear applications is often very small, and thus little disturbances in mass can affect the test results. The advantage of weighing is that it is the simplest method for measuring wear rates. The disadvantages are due to the impossibility of using this method when the component under study cannot be dismounted or also when material is transferred on the components during the wear progress. Furthermore, this method does not enable evaluations of the wear distribution over the specimen’s surface.
- Surface activation: in surface activation, the target surface is activated with radioactive rays before wear. After the wear progression, the surface is examined with a radioactive-ray spectrometer and the change in activity shows the amount of wear. The advantage of surface activation is the possibility of performing simultaneous measurements of wear rates of various parts. The disadvantage is that this method is inaccurate and it is difficult to ensure safety during the procedure.
- Ultrasonic reflectometry and phase interference [10,11]: real time measurements of wear can be performed by these methods. Ultrasonic reflectometry is commonly used in the field of non-destructive testing (NDT) for crack detection, wall thickness monitoring, and medical imaging. A sound wave is emitted through the specimen under study using a piezoelectric transducer. This waveform travels through the host medium at a constant speed and is either partially or fully reflected at an interface. The reflected wave is picked up by the same sensor; the signal is then amplified and digitized. If the speed that sound travels through a host medium is known as well as the time this takes, the thickness of the material can be established [10]. Also, ultrasonic phase-comparison techniques can be used [11]. The advantage of these methods is the very high resolution. In-fact, up to 1 μm dimensional changes can be detected. The main disadvantage deals with the requirement of highly specialized personnel.
- 2D digital image processing [12,13,14,15,16]: this method is based on computer vision and statistical learning systems to estimate the wear level, for example in manufacturing tools, in order to identify the time for their replacement. Binary images for each worn specimen’s image can be obtained by applying several pre-processing and segmenting operations. Then every worn region can be described by means of some geometrical descriptors (as for example eccentricity, extent and solidity). Based on the results obtained using a finite mixture model approach, different levels of wear can be detected (for example low, medium, and high wear level). The estimate of the wear level enables replacement of the tool when the wear level is located at the end of the medium class, preventing the tool from falling into the high class. An estimation of wear can also be done by comparing 2D profiles, measured on the components during the wear progress, by processing those profiles using digital image processing techniques, as in [15,16]. The advantage of this method is the simplicity and the possibility of taking pictures without dismounting the component. On the other hand, the method is slow and not very accurate.
- Manual thickness measurements, contact microscopy, and profilometry and surface detection [17,18,19,20,21,22]: these methods imply the use of callipers, comparators, or devices called contact or stylus profilometers, coordinated measuring machines (CMM) and atomic force or friction force microscopes (AFM/FFM). Stylus profilometers and CMM are measuring instruments able to detect the 2D profile of a specific section of the specimen (in the case of profilometers) and the 3D topography of the target surface (in the case of CMMs) by contacting the specimen’s surface with a sensing probe (a diamond stylus or metal balls). AFM/FFM microscopes are particular kinds of scanning force microscopes (SFM) sensitive to mechanical properties down to the nanometre scale. In particular, the contact mode atomic force microscope (AFM) is more suitable for the study of morphology and tribological effects on the micro-nanometre scale [21]. Such a technique is customarily employed for the characterization of solid thin films and is able to provide absolute values of the local kinetic or sliding friction. For example, in [22] an AFM provided tribological information about the morphology and the kinetic friction at the micro-nanometre scale. That information is evidenced when the sharp probe of an AFM—supported on an elastic cantilever beam—is moved laterally while contacting the sample surface. Such technique, named ‘friction force microscopy’ (FFM), is accomplished in a standard AFM by detecting the twisting movement of the cantilever beam, due to the lateral force acting on the tip, and provides information on the amount of sliding friction between the AFM tip and a local portion of the sample surface. Using those contact methods, measurements are made before and after wear and the two obtained surfaces are compared by numerical techniques to compute the loss of material. The main advantages regard the high accuracy of these instruments that can also provide the distribution of wear. As a disadvantage, they are slow and the specimen must be dismounted most of the time; they require the use of very expensive equipment and most of all the contact with the specimen’s surface can alter the wear characteristics.
- Optical microscopy, 2D profilometry, and 3D scanning [23,24,25]: these methods imply the use of optical non-contact profilometers, 3D scanners, and microscopes. Optical 2D profilometers and 3D scanners can work according to the principles of optical range-finding techniques as, for example, confocal microscopy, time of flight, laser or structured light triangulation, photogrammetry, interferometry, etc. 3D optical microscopes can work according to the principles of confocal microscopy, focus variation microscopy, scanning electron microscopy, fluorescence microscopy. Those methods show many advantages versus the prior ones. For example, they are very accurate, simple, and fast, they are able to provide the distribution of wear over the entire surface of the components, even in real time and for objects with very complex shapes. When the scanning devices are portable, the specimen does not need to be dismounted; the absence of contact does not alter the surfaces. Equipment are relatively cheap with respect to the prior ones, thanks to technological progress, and they enable very fast wear assessments. The only disadvantage is related to the 2D profilometry that is not a good solution when the specimen has a complex shape.

Indirect wear measuring methods are used only when dismounting or accessing specimens is not possible. Through those techniques, a set of resultant sources of data, which are caused by wear, are detected and measured.

In this paper, an innovative computer-aided methodology to detect and evaluate wear is proposed in the field of ‘digital tribology’. In particular, the aim of this study is to validate a new and more accurate method to assess wear on different samples by using cutting-edge 3D optical non-contact scanners. With respect to each other wear evaluation method, the proposed technique presents many advantages. First of all, with the use of 3D scanners, the errors due to inhomogeneity or liquid absorptions can be avoided and more accurate results can be achieved. In addition, the wear distribution over the sample’s surface can be assessed with a very good accuracy (up to 5–10 μm), even for objects with very complex shapes. The procedure enables non-invasive and non-contact measurements, preserving the topography and morphology of the target surface. The 3D digitization of the specimen can be done in real time and the digital wear assessment procedure is faster than all the prior ones; actually, it can be even automated by specific hardware and/or software. Dismounting the worn component can also be avoided most of the time.

## 2. Methods

### 2.1. Experimental Approach

**Figure 1:**
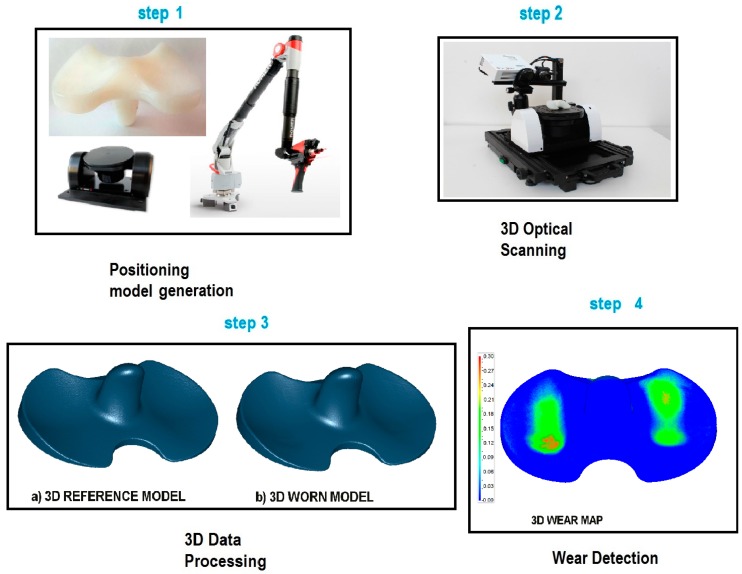
Experimental approach.

The proposed technique to detect and measure wear implies the use of optical non-contact 3D scanners for metrology and a wide range of applications including inspection and quality control. The digital procedure can be performed by following the steps according to the scheme in Figure 1.

The experimental approach here described is general purpose, as it explains how to perform the proposed procedure with any kind of 3D scanner and inspection software. Each step of the procedure is discussed, taking into account all the possible commercial available scanners and software or research prototypes. 

The first step (step 1) of the procedure consists of the creation of the positioning model, that is the reference system that the optical scanner employs to merge data from each frame into a full 3D digital model of the specimen. The positioning model allows complete automatic real-time reconstructions or manual alignments frame by frame. It can be generated by using four kinds of references: absolute reference systems, physical markers or targets, virtual markers, and geometrical features.

Physical markers are objects that can be easily detected by the optical devices and are often made by high reflective materials. When the markers are glued to the object and the scanning device is portable, the object can be moved through the scene without losing the reference and the link to the scanning device. 

When the scanning device has an absolute reference system, as in the case of scanning heads mounted on articulated arms or in the case of automated n-degrees of freedom desktop scanners with automated optical and mechanical calibrations [26,27], the use of physical markers can be avoided if the object does not move through the scene.

Virtual markers are reference points provided by the natural color features of the specimen and are used by the optical device to detect the displacement of the same object between the multiple frames during the scanning procedure. Geometrical attributes of the surfaces can be used by some devices to compare the scans frame by frame using best fit algorithms, in order to automatically or manually reconstruct the full 3D digital model of the specimen.

In the 3D optical scanning phase (step 2), 3D models of the worn and reference components under study are acquired and digitized. The reference model can be:

- The nominal CAD model of the specimen as it results from the design phase.
- The digitized 3D model of the specimen at the beginning of its useful life. In this second case, the model can be reconstructed from the real specimen before its exercise or from another specimen, identical to the worn one, before use.

The CAD model is useful in all the situations when neither the 3D model of the specimen before use, nor an identical new component, are available. In this case, the confidence level of the production cycle should be known to take into account the variability of the dimensions and shape of the CAD model from the real component after manufacturing.

Also, in the case of reconstruction of the reference model from another specimen, identical to the worn one before use, the knowledge of the level of confidence of the manufacturing process is useful. 

The best and more confident procedure to analyze and evaluate wear rates and distribution is to use the real specimen before its exercise to reconstruct the 3D reference model. 

The output 3D digital model is a point cloud or a triangular mesh. Before performing the final wear inspection, the optimization of the data by using mesh editing software is carried out (step 3). In this phase, it is crucial to improve the quality of the mesh or of the point cloud, taking care to preserve the original shape and dimension of the 3D model. To perform a good optimization of the 3D data, the following operations must be performed: detection and restoration of errors as non-manifold edges, correction of self-intersections, highly creased edges, spikes, small components, and small holes.

The digital wear inspection procedure (step 4) is performed by comparing the digitized 3D model of the worn specimen with the reference 3D model of the non-worn specimen. The procedure includes the following steps:

(a) The worn and the reference models must be aligned and superimposed to evaluate the deviations.
(b) The difference of volume between the worn and non-worn models can be calculated to evaluate the wear rates.
(c) The color 3D map of deviations is built, representing the wear distribution over the specimen’s surface.

The wear rate can be calculated as follows:

(1) $$\chi =\frac{V_{i}-V_{f}}{V_{i}}\;\%$$

where *V_i_* is the volume measured in the reference model and *V_f_* is the volume measured in the 3D model of the worn component. In addition, from the 3D wear maps, 2D sections and profiles can be extracted and studied. Furthermore, particular regions of interest can be investigated with deeper detail by measuring further geometrical wear parameters (wear depth, wear direction, etc.) 

### 2.2. Limits of the Experimental Procedure

Although the procedure can be considered validated by using certificated metrological scanners, whose statistical performance is known (repeatability, resolution, precision, accuracy), some issues can be identified. Sometimes, when the repeatability of the measurements is not known, it is necessary to digitize the surfaces many times, in order to obtain information about the repeatability of the procedure. 

Since the alignment procedure performed by registration software, based on distance minimizing criteria, can be another error source, the choice of the surface portions to use as reference for the alignment is also crucial. The right criteria to select the portions are different case by case, as they depend on many factors. First of all, the size of the worn portion is fundamental, as the alignment process should start by aligning three different regions not affected by wear; secondly, the shape of the component is important, as the geometric characteristics are used during the superimposition to find the best fit. Depending on the used scanner, some specimens with a shiny or semi-transparent surface could need the application of a matting coating, for example by means of spray powders. In this case, also the thickness of the powder layer must be taken into account in the measurements. Considering that the resolution and accuracy of optical 3D scanners commonly are not better than 5–10 μm, with such devices small values of roughness cannot be evaluated. 

### 2.3. 3D Optical Scanning and Devices

The 3D scanning procedure depends on the particular 3D scanner used in the application.

In wear evaluation applications, the most suitable devices are based on confocal laser scanning microscopy and triangulation, because they present the best performance in terms of resolution, precision, and accuracy.

Devices based on confocal laser scanning microscopy (CLSM or LSCM) use a technique which allows acquiring high-resolution in-focus images from various depths inside a specimen. This procedure is called optical sectioning and allows 3D reconstructions of the surface profile of opaque specimens and of the interior portion of non-opaque specimens [28,29]. Confocal laser scanners can be both desktop and portable.

Devices based on triangulation are able to digitally determine the location of each point on the surface of a target object by measuring angles from known points at each end of a fixed baseline. Each measured point can then be fixed as the third point of a triangle with one known side and two known angles. In passive triangulation or stereovision devices, no kind of electromagnetic radiation is projected on the scene by the scanning instrument, so that the unique radiation to be detected from the device is the ambient light reflected by the target specimen. In passive triangulation, two cameras whose relative positions and perspectives are known are used to capture stereo images. The main advantage of a stereovision system is the low cost of the components. The same principle can be applied also using three or more cameras instead of two. This method is not applicable to featureless surfaces.

In active triangulation methods, a light source is used to project a radiation onto the scene. The scene coverage can be achieved either by scanning light spots or line stripes expanded from a spot using a cylindrical lens, or by projecting structured light patterns of dots or lines onto the scanning area by specific grids. In active triangulation, one or more cameras can be used to capture the reflection of the light projected onto the object, in order to calculate the 3D shape [30,31,32]. 

## 3. Wear Characterization by Using 3D Optical Scanners

In this work, two different case studies are described. An industrial and a biomedical application of optical 3D scanners together with the proposed procedure are analyzed. In the first case (test #1), the wear resistance of some mixing blades mounted on a two-star planetary concrete mixer is assessed. In the second case (test #2), the loss of material of some knee joint prostheses is evaluated after wear tests onto a knee mechanical simulator.

### 3.1. Test 1: Wear Characterization of Mixing Blades

In this first test, the wear resistance of mixing blades used in planetary concrete mixers is evaluated, by using a 3D optical scanner [33,34] and two other methods commonly used in prior similar applications. In particular, in this case, the wear progress is usually assessed on the basis of the thickness reduction of some characteristic points on the blade front profile. The three different wear assessment methods used in this study are: manual thickness measurements, contact profilometry, and optical 3D scanning. 

This test involved three different blades mounted on the same mixer. Each blade underwent the same tests described hereinafter and the results are presented as mean values.

**Figure 2:**
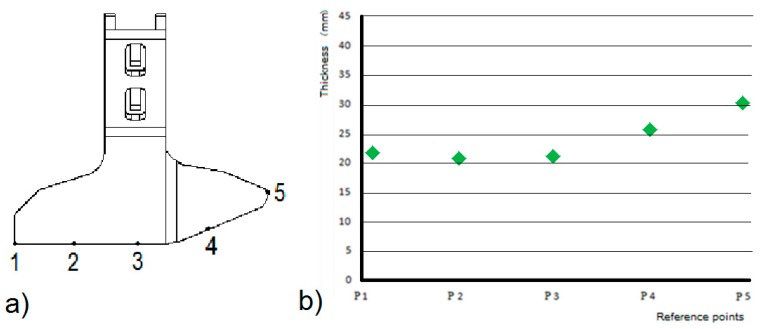
Reference points (**a**) and mean thickness of the worn material (**b**).

Five reference points were picked at the blades’ front profile, as shown in Figure 2 and the test started by measuring the thickness at those points by callipers and comparators. The result in terms of thickness of worn material is represented in Figure 2.

**Figure 3:**
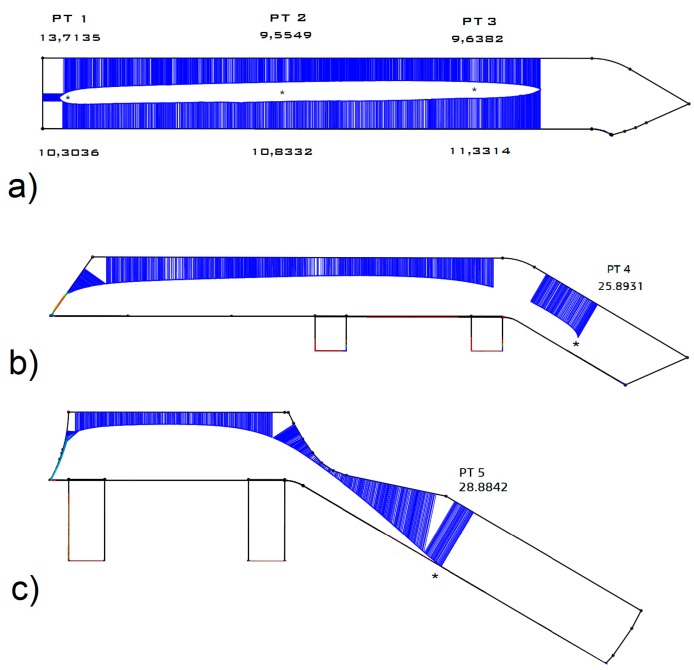
Mean thickness of the worn profiles: (**a**) points 1, 2, 3; (**b**) point 4; (**c**) point 5.

The measurements were repeated by using a contact stylus profilometer Linear Height LH-600E/EG—Series 518 (Mitutoyo Corp., Kawasaki, Japan). The results are shown in Figure 3, where the thickness of the worn profile is expressed in millimetres on the bottom and on the top of the worn section. In the pictures, both the section of the CAD reference model and the section of the worn model reconstructed by the 2D profilometer are represented. As one can observe, the total amount of material loss agrees with the manual measurements, but in this case the 2D wear distribution over the blade’s profile can be displayed.

**Figure 4:**
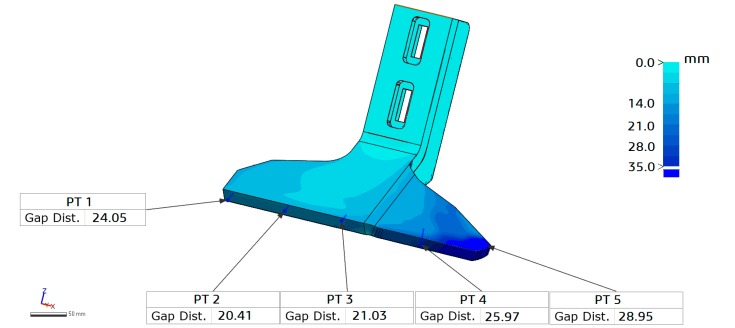
Blade’s 3D wear distribution map (mean values).

The last test was performed by means of an ultra-accurate 3D laser scanner: HandySCAN 700 (Creaform Inc., Levìs, QC, Canada). In this case, the method implied the following steps:

(a) The worn model was scanned by HandySCAN 700 and the full 3D model was automatically and real time reconstructed by the scanning software.
(b) The worn model was superimposed and aligned to the CAD reference model with the mesh editing and modeling software Geomagic Control X (3D Systems, Rock Hill, SC, USA).
(c) The 3D color map of the wear distribution was built.
(d) The wear distribution was evaluated by comparison between the volumes calculated on the CAD and worn model and the reference points were checked to get the value of the gap distance, representing the material loss. The result is shown in Figure 4.

The 3D laser scanner used to create the digitized 3D models of the worn blades was a portable real time device, able to reconstruct the full worn object in a few minutes, by projecting seven laser crosses onto the scene. In this case, the time from step 1 to 2 was three minutes for each blade. Before scanning, the blade was cleaned to remove the remaining concrete. Then physical targets (high reflective white circles with black contours) were applied over the entire surface of the blades to generate the positioning model (step 1). The scanner was calibrated before starting the acquisition by an automatic procedure, which lasted 20 seconds, in order to assure the achievement of the best performance (30 μm accuracy). The software used for the acquisition was VXelements 5.1 SR2 (Creaform Inc., Levìs, QC, Canada). The scanning procedure was performed at 0.2 mm resolution and the models of the worn blades were reconstructed in real time (step 2). The output of the scanner is a polygonal model or mesh, where the polygons are triangles. An automatic mesh optimization tool was enabled during the scanning procedure (mesh and boundaries optimization, decimation, partial hole filling, and isolated patche removal) to simplify the next step of the procedure (step 3). The data optimization procedure (step 3) was performed taking care to preserve the original shape and volume of the rough 3D data through the software VXmodel 5.1 SR2. 

After the mesh optimization, the worn blades’ 3D models were compared with the corresponding reference nominal 3D CAD model (step 4) by using Geomagic Control X (3D Systems, Rock Hill, SC, USA). The time from step 3 to 4, performed by specialized researchers, was 1.5 hours for each blade.

### 3.2. Test 2: Wear of a Knee Joint

A knee prosthesis is a complex medical device that is used on patients when they can no longer stand the pain due to their arthritis. During their life, the polyethylene prostheses could be affected by wear so that preclinical wear tests are necessary to simulate the motion and interaction between the contact materials [35]. In this test, two different wear evaluation methods are applied and compared on ultra-high-molecular-weight-polyethylene (UHMWPE) tibial meniscus knee prostheses. 

The amount of mass loss was assessed on three components after two million cycles wear tests onto the knee joint mechanical simulator ‘three-plus-one’ (Shore Western Mfg., Monrovia, CA, USA). In particular, considering that in vitro studies the wear can be measured quite accurately before, during, and a after the simulation using the gravimetric method, in this test the wear was evaluated by comparing the weighing and the new digital procedure. The optical device used in this test was the structured light 3D scanner ScanRider 1.2 (V-GER SRL, Bologna, Italy) [36]. In this study, three worn samples and an unworn check reference model of the same batch were considered. The reference check model was a specimen, identical to the worn ones before use. Thus, four 3D digital models have been reconstructed by the scanner. The specimens have been weighed before scanning, by means of an ultraprecise scale (Sartorius Cubis MSE 225 S-000-DU, Goettingën, Germany).

**Figure 5:**
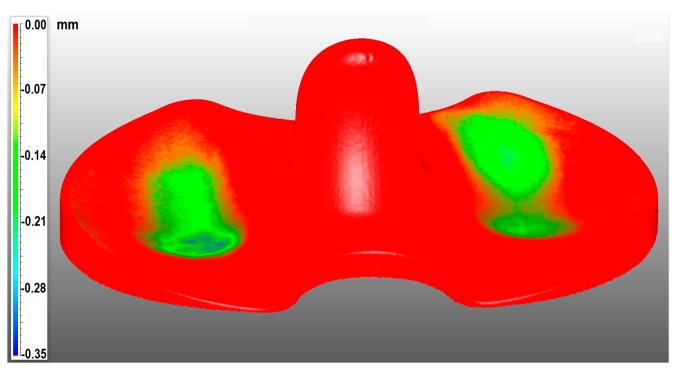
Tibial meniscus #2 3D wear map.

Then, the worn samples and the check have been 3D scanned and the full digital models superimposed, aligned, and digitally inspected using the software VXinspect (Creaform Inc., Levìs, QC, Canada). The alignment was performed by taking as a reference the unworn portions of the contact area of the tibial meniscus. Those unworn parts are localized, especially at the edges of the meniscus. The resulting 3D wear map of component #2 is shown in Figure 5.

The 3D scanner used to create the digitized 3D models of the worn and check components was an automated device allowing full 3D reconstructions without any human intervention. In this case, it was not needed to apply physical targets as the positioning model is provided by the automatic mechanism reference system, so step 1 was not performed. The scanning time (step 2) was one minute per meniscus. The scanner was calibrated before starting the acquisition by an automatic procedure, which lasted 25 seconds, in order to assure the achievement of the best performance (10 μm accuracy). The software used for the acquisition was SpaceRider (V-GER SRL, Bologna, Italy) and the output of the scanner was a triangular mesh. The data optimization procedure (step 3) was performed taking care to preserve the original shape and volume of the rough 3D data through the software VXmodel 5.1 SR2. After the mesh optimization process, the worn meniscus models were compared with the corresponding reference check model (step 4) by using the software VXinspect (Creaform Inc., Levìs, QC, Canada). The time from step 3 to 4, performed by specialized researchers, was one hour for each meniscus.

## 4. Results and Discussion

Regarding the first test, one can observe that the total amount of material loss found with the three methods are comparable, as shown in Table 1.

In particular, the table shows that the maximum difference between the results from the 2D contact profilometer and the 3D scanner is 80 μm. This deviation is due partially to the error of accuracy of the instruments (1.5 μm for the contact profilometer; 30 μm for the 3D scanner) and partially to the error of positioning of the profilometer on the exact point of the blade. Thus, the positioning of the profilometer’s probe is a potential error source. In addition, in many cases the direct contact must be avoided in order to preserve the surface roughness and morphology. This problem can be overcome by using optical devices. Furthermore, the proposed method, based on 3D optical scanners, enables the gathering of a complete scenario of the wear phenomenon on the component in the 3D space. In fact, from the 3D wear maps all the wear parameters can be deduced. 

Regarding the case of the knee prostheses, considering a uniform distribution of density, a variance in mass between the unworn and worn models was obtained as reported in Table 2. As one can observe, there are no significant differences between the results of the two methods. 

In any way, the digital procedure allowed again not only to assess the amount of material loss but also to get the real 3D wear distribution over the surface of the specimen, that is very useful to study the behavior of the prosthesis during the contact in the knee joint, to improve the design and manufacturing.

With respect to each other wear evaluation method, the proposed technique presents many advantages. With respect to the weighing method, with the use of 3D scanners the errors due to inhomogeneity or liquid absorptions can be avoided and more accurate results can be achieved. In addition, with gravimetric tests only the value of the mass wear rate can be calculated without any attention to the wear distribution over the sample’s surface. With respect to manual thickness measurements by means of callipers or also contact profilometers or contact CMM, the advantage of using 3D optical scanners lies in the fact that non-invasive measurements can be performed and the topography and morphology of the target surface can be preserved. With respect to surface activation, 3D scanners enable more accurate results. Using ultrasonic reflectometry, phase interference and microscopy the results are very accurate but the procedure can be more time consuming.

Nevertheless, there exist some limitations and error sources regarding the use of the proposed technique for wear assessment. For example, the alignment procedures can introduce errors due to a wrong choice of the reference surface portions. In addition, with some 3D optical scanners, specimens with a shiny or semi-transparent surface cannot be detected without applying a matting coating. Regarding the scale limitations, the resolution and accuracy of optical 3D scanners are commonly not better than 5–10 μm so that small values of roughness cannot be evaluated.

## 5. Conclusions

In this paper, some of the present wear evaluation methods have been showed and discussed. Then an innovative procedure to detect and evaluate wear, in the field of digital tribology, has been presented. This method can be performed by using innovative 3D optical scanners, made available by the technological progress and innovation. This procedure is applicable to any kind of worn specimen and provides very accurate results, depending on the metrological performance of the used devices. For this reason, some of the most suitable 3D optical non-contact metrological devices have been discussed. The novelty of the proposed digital procedure is that it is simpler, faster, and in many cases more accurate with respect to all the other wear evaluation methods and it allows the reconstruction and display of a 3D wear map of the components under study. Thus, the real distribution of the phenomenon on the different portions of the object is visible and quantifiable and all the wear parameters are extractable from the 3D map.

The procedure presented in this work shows the general approach to take into account for wear evaluations. Going further, the standard steps described in the paper, as some steps can be different case by case, the correct and fruitful application of the procedure relies also on the ability and experience of the researcher on digital matters.

## Figures and Tables

**Table 1:** Comparison of Results in Test 1

Reference Point No.	Manual Thickness Meas. (mm) (Mean Value)	2D Contact Profile Meas. (mm) (Mean Value)	3D Scanning Meas. (mm) (Mean Value)
#1	23.31	24.02	24.05
#2	20.52	20.39	20.41
#3	21.03	20.97	21.03
#4	25.53	25.89	25.97
#5	30.10	28.88	28.95

**Table 2:** Comparison of Results in Test 2

Sample No.	Mass Deviation From Gravimetric Tests (mg)	Mass Deviation From 3D Wear Maps (mg)
#1	13.05	12.30
#2	6.10	6.30
#3	7.50	8.80
